# Structure and specificity of the Type VI secretion system ClpV-TssC interaction in enteroaggregative *Escherichia coli*

**DOI:** 10.1038/srep34405

**Published:** 2016-10-04

**Authors:** Badreddine Douzi, Yannick R. Brunet, Silvia Spinelli, Valentine Lensi, Pierre Legrand, Stéphanie Blangy, Anant Kumar, Laure Journet, Eric Cascales, Christian Cambillau

**Affiliations:** 1 Architecture et Fonction des Macromolécules Biologiques (AFMB, UMR 6098), Centre National de la Recherche Scientifique (CNRS), Campus de Luminy, Case 932, 13288 Marseille Cedex 09, France; 2 Architecture et Fonction des Macromolécules Biologiques (AFMB, UMR 6098), Aix-Marseille Univ, Campus de Luminy, Case 932, 13288 Marseille Cedex 09, France; 3 Laboratoire d’Ingénierie des Systèmes Macromoléculaires (LISM, UMR 7255), Institut de Microbiologie de la Méditerranée (IMM), Centre National de la Recherche Scientifique (CNRS), Aix-Marseille Univ, 31 chemin Joseph Aiguier, 13402 Marseille Cedex 20, France; 4 Synchrotron Soleil, L’Orme des Merisiers, Saint-Aubin - BP 48, 91192 Gif-sur-Yvette Cedex, France

## Abstract

The Type VI secretion system (T6SS) is a versatile machine that delivers toxins into either eukaryotic or bacterial cells. It thus represents a key player in bacterial pathogenesis and inter-bacterial competition. Schematically, the T6SS can be viewed as a contractile tail structure anchored to the cell envelope. The contraction of the tail sheath propels the inner tube loaded with effectors towards the target cell. The components of the contracted tail sheath are then recycled by the ClpV AAA^+^ ATPase for a new cycle of tail elongation. The T6SS is widespread in Gram-negative bacteria and most of their genomes carry several copies of T6SS gene clusters, which might be activated in different conditions. Here, we show that the ClpV ATPases encoded within the two T6SS gene clusters of enteroaggregative *Escherichia coli* are not interchangeable and specifically participate to the activity of their cognate T6SS. Here we show that this specificity is dictated by interaction between the ClpV N-terminal domains and the N-terminal helices of their cognate TssC1 proteins. We also present the crystal structure of the ClpV1 N-terminal domain, alone or in complex with the TssC1 N-terminal peptide, highlighting the commonalities and diversities in the recruitment of ClpV to contracted sheaths.

The Type VI secretion system (T6SS) is a multi-protein complex widely distributed in Gram-negative bacteria with an over-representation in Proteobacteria and Bacteriodetes responsible for the transport and delivery of effector toxins into target cells^1^,^2^,^3^,^4^. The activities and molecular targets of the T6SS effectors correlate with the specific needs of the bacterium in its environmental niche. In most bacteria, the T6SS confers a competitive advantage in multi-species environments, as it delivers anti-bacterial toxins with peptidoglycan hydrolase, phospholipase or DNase activity into target bacterial cells^5^,^6^,^7^,^8^. The T6SS thus regulates bacterial populations and facilitates colonization of the environment^9^. In addition to its role in the bacterial warfare, a few T6SS have been shown to secrete toxins that are active in eukaryotic cells, such as proteins that interfere with the actin or tubulin assembly pathways^10^,^11^,^12^,^13^. The T6SS comprises 13 conserved and essential components named TssA to TssM^14^,^15^. These core-components assemble two sub-complexes^15^,^16^,^17^. The first sub-complex is evolutionarily, structurally and functionally similar to the tail structures of contractile bacteriophages^14^,^18^,^19^. It is constituted of a ~600 nm-long inner tube made of Hcp hexamers stacked on each other, and wrapped into a sheath-like structure^20^,^21^. The sheath-like structure is composed of rows of heterodimers of TssB and TssC (VipA and VipB in *Vibrio cholerae*), which assemble into an extended, high-energy conformation^16^,^18^,^20^,^22^,^23^,^24^. The Hcp inner tube is tipped by the VgrG/PAAR complex, that serves as puncturing device for the penetration of the target cell, as well as adaptor complex for effectors^25^,^26^,^27^,^28^,^29^. The VgrG spike protein is also part of the assembly platform composed of the TssEFGK proteins^30^,^31^. This platform - or baseplate - controls the polymerization of the tube/sheath structure and probably initiates sheath contraction^31^,^32^. This platform is tightly attached to the cell envelope through multiple contacts with components of the second sub-complex, called membrane complex^31^,^33^. The membrane complex is composed of three proteins: the inner membrane TssL and TssM proteins and the TssJ outer membrane lipoprotein^34^,^35^,^36^,^37^,^38^. These three proteins are present in 10 copies each and assemble a 1.7-MDa complex that crosses the cell envelope and delimits a channel for the passage of the inner tube during sheath contraction^39^. Once in contact with a target cell, or when the cell senses an attack by a competitor, the T6SS assembles the tubular structure and the sheath contracts, hence propelling the inner tube/spike towards the target cell and delivering toxins^20^,^40^. While the membrane complex is stable and can be reused for multiple injections^39^, the contracted sheath is disassembled by a dedicated AAA^+^ ATPase of the Clp family, ClpV, and recycled for a new assembly and injection cycles^22^,^23^,^41^. In most T6SS, ClpV is recruited to the contracted sheath via interactions with the N-terminal helix of the TssC subunits that is thought to be accessible only in the contracted conformation^22^,^23^,^41^,^42^,^43^. The N-terminal helix of TssC accommodates into a cleft constituted of charged residues located at the interface of two helices, called H1 and H2, at the N-terminus of ClpV^41^,^43^. However, in a subset of T6SS, the cleft is composed of uncharged residues, and the ClpV-TssC interaction is mediated or stabilized by the TagJ accessory protein^43^.

Interestingly, most bacteria encode several copies of T6SS gene clusters in their genomes^1^,^2^,^3^,^44^. These clusters are usually responsive to different environmental cues and hence subjected to different regulatory mechanisms^45^. It is thought that this diversity is responsible for the activation of distinct T6SS machineries in different conditions. However, little is known on how the subunits select their cognate machineries when multiple paralogous T6SS are present in the same cell. Enteroaggregative *Escherichia coli* strain 17-2 encodes two T6SS gene clusters of the T6SS-1 and T6SS-3 sub-families^44^, and it has been shown that the inner tube component Hcp encoded by the T6SS-1 cluster (*sci-1*) specifically interacts with the sheath component TssB1, but not with TssB2, which is encoded by the T6SS-3 cluster (*sci-2*), demonstrating specificity during assembly of the T6SS tails^21^. Here, we report that specificity also applies to disassembly of the contracted sheath. We demonstrate that over-production of ClpV2 cannot compensate for the absence of ClpV1 and cannot restore functionality of the T6SS-1 (and vice versa). We further provide evidence that ClpV1 specifically interacts with the N-terminal helix of TssC1 and not with that of TssC2. We then report the crystal structure of the ClpV1 N-terminal domain alone and in complex with the TssC1 N-terminal helix. Noteworthy, the crystal structure of the complex differs significantly from what was reported for ClpV-VipB complex from *V. cholerae*^41^. Our results suggest an alternative mode of binding between TssC1 and ClpV1, as the cleft in ClpV1 is essentially composed of uncharged residues but does not require TagJ to efficiently bind to the TssC1 N-terminal helix.

## Experimental Procedures

### Bacterial strains and media

The *Escherichia coli* K-12 DH5α, BTH101, W3110 and BL21(DE3) pLysS strains were used for cloning procedures, bacterial two-hybrid analyses, co-immunoprecipitations and protein production, respectively. Strain W3110 pUA66-*rrnB* (Kan^R^, GFP^+^)^46^ was used as prey in anti-bacterial competition experiments. Cells were grown in Lysogeny broth (LB), Sci-1-inducing medium (SIM) or Dulbelcco modified Eagle medium (DMEM), as specified. Plasmids were maintained by the addition of ampicillin (100 μg/mL), chloramphenicol (40 μg/mL) or kanamycin (50 μg/mL).

### Plasmid construction for *in vivo* studies

Plasmids used in this study are listed in Supplemental Table S1. Polymerase Chain Reactions (PCR) were performed using a Biometra thermocycler using the Q5 high fidelity DNA polymerase (New England BioLabs). Custom oligonucleotides, listed in Supplemental Table S1, were synthesized by Sigma Aldrich. Enteroaggregative *E. coli* 17-2 chromosomal DNA was used as a template for all PRCs. The amplified DNA fragments correspond to the full-length ClpV1 (EC042_4530, GI: 284924251), ClpV2 (EC042_4577, GI: 284924293), TssC1 (EC042_4525, GI: 284924246) and TssC2 (EC042_4562, GI: 284924279) proteins, as well as the N-terminal domains of ClpV1 (residues 1–163) and ClpV2 (residues 1–147). Plasmids were engineered by restriction-free cloning^47^ as previously described^35^. Briefly, genes of interest were amplified with oligonucleotides introducing extensions annealing to the target vector. The double-stranded product of the first PCR was then been used as oligonucleotides for a second PCR using the target vector as template. Deletion of TssC1 and TssC2 N-terminal helices as well as point mutations have been obtained by site-directed mutagenesis. All constructs have been verified by restriction analysis and DNA sequencing (Eurofins, MWG).

### Bacterial two-hybrid assay

The adenylate cyclase-based bacterial two-hybrid technique^48^ was used as previously published^49^. Briefly, compatible vectors producing proteins fused to the isolated T18 and T25 catalytic domains of the *Bordetella* adenylate cyclase were transformed into the reporter BTH101 strain and the plates were incubated at 30 °C for 24 hours. Three independent colonies for each transformation were inoculated into 600 μL of LB medium supplemented with ampicillin, kanamycin and IPTG (0.5 mM). After overnight growth at 30 °C, 10 μL of each culture were spotted onto LB plates supplemented with ampicillin (100 μg/mL), kanamycin (50 μg/mL), IPTG (0.5 mM) and Bromo-Chloro-Indolyl-Galactopyrannoside (X-Gal, 40 μg/mL) and incubated for 16 hours at 30 °C. The experiments were done at least in triplicate and a representative result is shown.

### Co-immunoprecipitations

100 mL of W3110 cells producing the proteins of interest were grown to an absorbance at λ = 600 nm (*A*_600_) ~ 0.4 and the expression of the cloned genes were induced with AHT (0.1 μg/mL) and L-arabinose (0.2%) for 45 min. The cells were harvested, and the pellets were resuspended in 20 mM Tris-HCl pH 8.0, 100 mM NaCl, 30% sucrose, 1 mM EDTA, lysozyme 100 μg/mL, DNase 100 μg/mL, RNase 100 μg/mL supplemented with protease inhibitors (Complete, Roche) to an *A*_600_ of 80 and incubated on ice for 20 min. Cells were lysed by three passages at the French Press (800 psi) and lysates were clarified by centrifugation at 20,000 × *g* for 20 min. Supernatants were used for co-immunoprecipitation using anti-FLAG M2 affinity gel (Sigma-Aldrich). After 3 hours of incubation, the beads were washed three times with 1 mL of 20 mM Tris-HCl pH 8.0, 100 mM NaCl, 15% sucrose, resuspended in 25 μL of Laemmli loading buffer, boiled for 10 min and subjected to SDS-PAGE and immunodetection analyses.

### Anti-bacterial competition assay

Antibacterial competition growth assays were performed as previously described, in Sci-2-inducing^40^ or Sci-1-inducing^29^ conditions. The wild-type *E. coli* strain W3110 bearing the kanamycin-resistant GFP^+^ pUA66-*rrnB* plasmid^46^ was used as prey.

### Plasmid construction for *in vitro* studies

The DNA sequence encoding the ClpV1 N-terminal domain (ClpV1-Nt, Gly1 to Leu163) was cloned into the pETG-20A expression vector using standard Gateway procedures to yield pETG20A-ClpV1-Nt. The resulting construction allows the production of ClpV1-Nt fused to an N-terminal Thioredoxin followed by a 6 × His tag and a Tobacco Etch Virus (TEV) cleavage site. Primers used for genes amplification are shown in Supplemental Table S1.

### Peptide synthesis, protein production and characterization

*E. coli* BL21 (DE3) pLysS cells carrying the pETG20A-ClpV1-Nt plasmid were grown at 37 °C in TB medium (1.2% peptone, 2.4% yeast extract, 72 mM K_2_HPO_4_, 17 mM KH_2_PO_4_, and 0.4% glycerol). Expression of *clpV1-Nt* was induced at *A*_600_ = 0.6 with 0.5 mM IPTG for 18 hours at 25 °C. Cells were then resuspended in lysis buffer (50 mM Tris-HCl pH 8.0, 300 mM NaCl, 1 mM EDTA, 0.5 mg/mL lysozyme, 1mM phenylmethylsulfonyl fluoride), submitted to several freeze-thawing cycles and sonicated after the addition of 20 μg/mL DNase and 20 mM MgCl_2_. Pellet and soluble fraction were separated by centrifugation for 30 min at 16,000 × *g*. The soluble fraction containing ClpV1-Nt was loaded onto a 5-mL Ni^2+^ affinity column (HisTrap™ FF) using an ÄKTA Explorer apparatus (GE healthcare) and the immobilized proteins were eluted in 50 mM Tris-HCl pH8.0, 300 mM NaCl supplemented with 250 mM imidazole. The protein solution was desalted on a HiPrep 26/10 column (Sephadex^TM^ G-25, Amersham Biosciences), and ClpV1-Nt was obtained by cleavage using 2 mg of TEV protease for 18 hours at 4 °C and collected in the flow-through of a 5-mL Nickel column. The protein was concentrated using the centricon technology (Millipore, 10-kDa cut-off). After concentration, the soluble ClpV1-Nt protein was passed through a Sephadex 200 26/60 column pre-equilibrated with 25 mM Tris-HCl pH7.5, 100 mM NaCl, 5% Glycerol. A similar procedure was applied for the ClpV2-Nt domain but the protein was insoluble and could not be purified.

Peptides corresponding to the α-helices from TssC1 and TssC2 were synthesized by Genscript (TssC1 (residues 23–35), KKW-DSVYASLFEKINL-KK; TssC2 (residues 15–29), ATDDCLEEIINNTRA). Due to its hydrophobic nature, the TssC1 N-terminal peptide was flanked by two lysines to improve its solubility and by an additional tryptophane to follow it during purification.

### Microscale thermophoresis (MST) experiments

Microscale thermophoresis experiments were performed using a Monolith NT.115 apparatus (NanoTemper). ClpV1-Nt was labeled with the blue-fluorescent dye NT-495-NHS (NanoTemper) and the buffer was exchanged for the assay buffer (25 mM Tris-HCl pH 7.5, 200 mM NaCl, glycerol 5%, 0.05% Tween-20) using a Nap5 column (GE Healthcare). Titrations were conducted with a constant 200 nM fluorophore-labeled ClpV1-Nt against up to 500 μM TssC N-terminal peptides in hydrophilic-coated capillaries. Each data point was measured in triplicate. Single-site fitting was performed using the NanoTemper data analysis software.

### Crystal structures determination

ClpV1-Nt was purified to homogeneity and the protein crystallized spontaneously during gel filtration. ClpV1-Nt crystals belong to space group P2_1_2_1_2_1_ with cell dimensions a = 40.9Å, b = 58.7 Å, c = 65.6 Å (Supplemental Table S2). A complete data set was collected at the Soleil synchrotron (Saint-Aubin, France) at 2 Å. Data were integrated with XDS^50^ and reduced with XSXALE^50^ (Supplemental Table S2). The structure was solved by soaking crystals in a 0.5 M NaI and 0.5 M of CsI solution followed by data collection at 1.77-Å wavelength, allowing remote SAD phasing. The cesium/iodine positions were defined with SHELXC and SHELXD^51^ and subsequently used for SAD phasing using PHASER^52^. These phases were improved by solvent flattening and histogram matching using the program PARROT^53^, resulting in interpretable maps from which the BUCCANEER program^54^ was able to build an initial model. The model was completed and corrected manually with COOT^55^ and refined with autoBUSTER^56^ (Supplemental Table S2).

The ClpV1-Nt/TssC1 peptide complex was obtained by mixing the purified ClpV1 N-terminal domain with the peptide at a 1:4 molar ratio in 20 mM Tris-HCl pH8.0, 100 mM NaCl, 5% Glycerol and concentrated using the centricon technology (Millipore, kDa cut-off of 10) to 2 mg/mL. Crystals were obtained in one condition (0.2 M imidazole, malate pH 6.0, 8% w/v PEG 4000) using the STURA screen (Molecular dimensions) and diffracted at 2.5-Å resolution. The structure was solved by molecular replacement using the structure of ClpV1-Nt as starting model. The model was completed and corrected manually with COOT^55^ and refined with autoBUSTER^56^ (Supplemental Table S2).

#### Data deposition

The EAEC ClpV1-Nt and ClpV1-Nt/TssC1 peptide X-ray structures have been deposited in the Protein Data Bank (PDB) under accession numbers 4HH5 and 4HH6, respectively.

## Results

### ClpV1 and ClpV2 are not interchangeable

**Figure 1:**
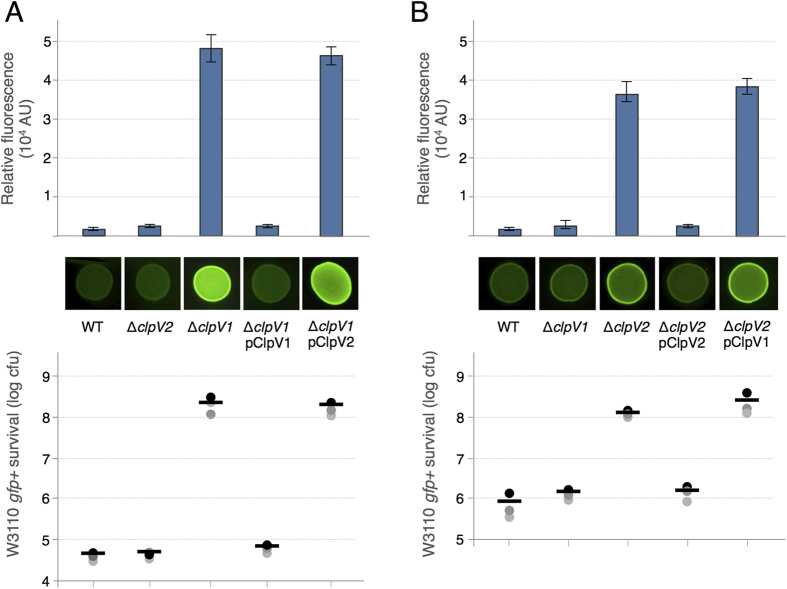
ClpV1-ClpV2 interchangeability. (**A**) Sci-1-dependent antibacterial growth inhibition. Prey cells (W3110 *gfp*^+^, kan^R^) were mixed with the indicated attacker cells, spotted onto *sci-1*-inducing medium (SIM) agar plates and incubated for 4 hours at 37 °C. (**B**) Sci-2-dependent antibacterial growth inhibition. Prey cells (W3110 *gfp*^+^, kan^R^) were mixed with the indicated attacker cells, spotted onto Dulbecco’s modified Eagle Medium (DMEM) agar plates and incubated for 4 hours at 37 °C. The image of a representative bacterial spot is shown and the relative fluorescent levels (in arbitrary units, AU) are indicated in the upper graph. The number of recovered *E. coli* prey cells is indicated in the lower graph (in log_10_ of colony-forming units (cfu)). The circles indicate values from three independent assays, and the average is indicated by the bar.

The enteroaggregative *E. coli* Sci-1 and Sci-2 T6SS are both involved in bacterial competition^29^,^40^. However, these two T6SS are active in different laboratory conditions (minimal medium for the Sci-1 T6SS and modified Eagle medium for the Sci-2 T6SS^57^,^58^). To test whether the two ClpV proteins, ClpV1 (EC042_4530, GI: 284924251) and ClpV2 (EC042_4577, GI: 284924293), respectively encoded by the *sci-1* and *sci-2* gene clusters are interchangeable, we engineered ∆*clpV1* and ∆*clpV2* strains and complementation vectors (pIBA-ClpV1 and pIBA-ClpV2). The different combinations were tested for their ability to confer a growth advantage in Sci-1- or Sci-2-active conditions against *E. coli* K-12. Figure 1 shows that while *clpV1* and *clpV2* trans-expression restores the growth advantage of the ∆*clpV1* and ∆*clpV2* mutant strains respectively, over-production of ClpV2 does not restore ∆*clpV1* defects, and vice versa. From this experiment, we concluded that the ClpV1 and ClpV2 ATPase are not interchangeable and we hypothesized that this specificity could be imputable to specific contacts with partners.

### ClpV specificity is dictated by ClpV-TssC complex formation

**Figure 2:**
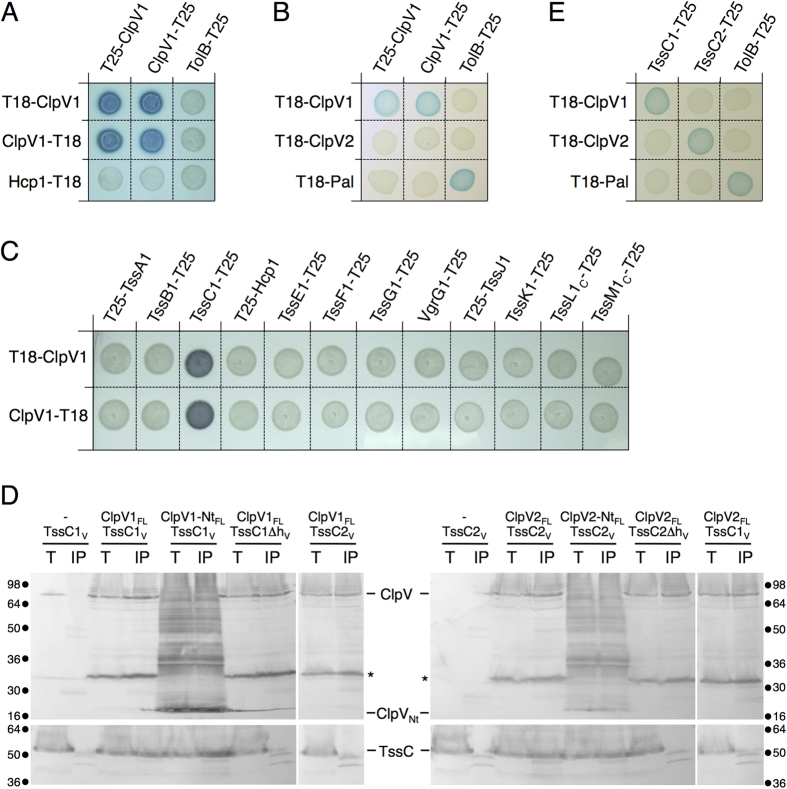
Interaction of the ClpV N-terminal domain with its cognate TssC protein. (**A–C,E**) Bacterial two-hybrid assay. BTH101 reporter cells producing the indicated proteins or domains fused to the T18 or T25 domain of the *Bordetella* adenylate cyclase were spotted on X-Gal indicator plates. The blue color of the colony reflects the interaction between the two proteins. (**D**) Co-immunoprecipitation assay. The soluble lysate from 10^11^ *E. coli* K-12 W3110 cells producing the indicated proteins (_FL_, FLAG-tagged; _V_, VSVG-tagged) were subjected to immune precipitation on anti-FLAG-coupled agarose beads. The immunoprecipitated material was subjected to 12.5%-acrylamide SDS-PAGE and immunodetected with anti-FLAG (upper panel) and anti-VSVG (lower panel) monoclonal antibodies. Molecular weight markers (in kDa) are indicated.

To identify ClpV1 partners, we performed a systematic bacterial two-hybrid analysis. ClpV1 was fused to the N- or C-terminus of the *Bordetella pertussis* Cya T25 domain and these protein fusions were tested as baits against preys comprising fusion of the T18 domain to the individual T6SS core components. Growth on reporter plates demonstrated that ClpV1 interacts with itself (Fig. 2A), in agreement with the ability of AAA^+^ ATPases to assemble hexameric structures^22^,^59^. However, ClpV1 does not interact with ClpV2 suggesting that these two proteins cannot assemble hetero-hexamers (Fig. 2B). In addition, ClpV1 interacts with TssC1 (EC042_4525, GI: 284924246), one of the sheath components (Fig. 2C). The ClpV1-TssC1 interaction was further confirmed by co-immunoprecipitation (Fig. 2D). The ClpV-TssC interaction has already been reported in *V. cholerae* and *P. aeruginosa* and involves contacts between the N-terminal domain of ClpV and an N-terminal helix of TssC^22^,^41^,^43^. Similarly, co-immunoprecipitation assays showed that the N-terminal domain of ClpV1 (ClpV1-Nt, residues 1–163) is sufficient to mediate the interaction with TssC1, while a deletion of the TssC1 N-terminal helix (deletion of residues 23–25, TssC1∆h) abolishes ClpV1-TssC1 complex formation (Fig. 2D, left panel). Identical results were obtained for ClpV2 and the Sci-2-encoded TssC2 protein (Fig. 2D, right panel). We then tested cross-interaction between the ClpV N-terminal domains and TssC proteins. Fig. 2D (right lanes in left and right panels) shows that each ClpV N-terminal domain interacts only with the TssC encoded within the same gene cluster. The lack of cross-interaction between the ClpV and TssC proteins from the two T6SSs was further confirmed by bacterial two-hybrid (Fig. 2E). From these results, we concluded that specificity determinants control ClpV-TssC complex formation and we hypothesized that these specificity determinants should be located within the TssC N-terminal helices. In support of this hypothesis, a sequence alignment of TssC1 and TssC2 showed that the two proteins share high level of homologies except in the N-terminal region that is, in average, less conserved (18% identity within the 55 N-terminal residues compared to 38% identity on full-length sequences, Supplemental Figure S1).

**Figure 3:**
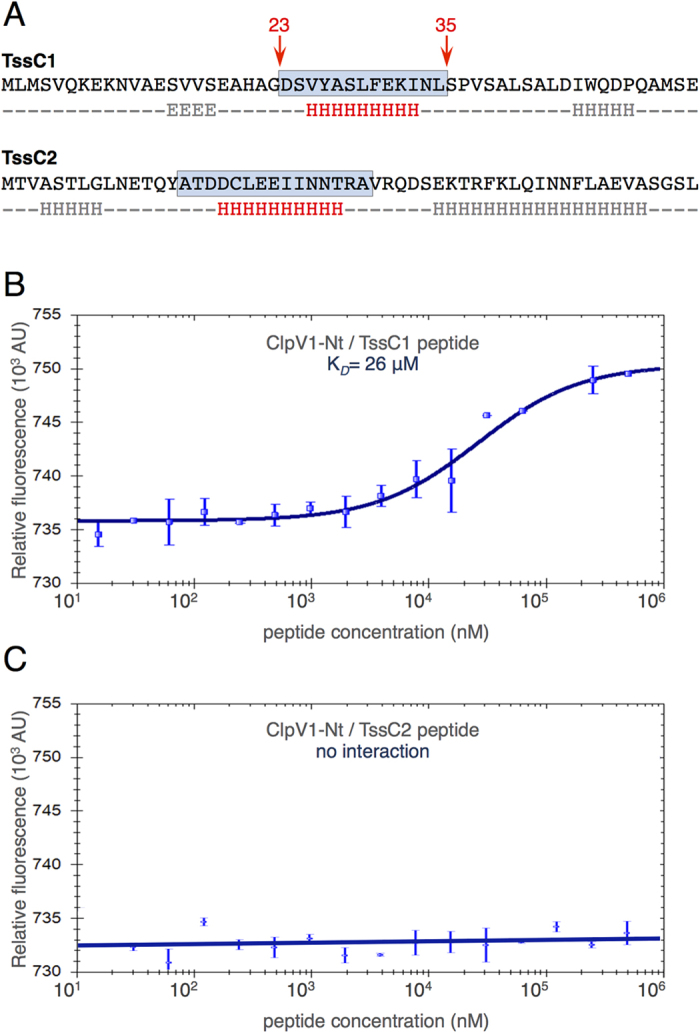
The TssC1 N-terminal helix specifically binds to the ClpV1 N-terminal domain. (**A**) Sequences of the TssC1 and TssC2 N-terminal regions. The secondary structure predictions (using JPRED^64^) are indicated under the sequence, and the TssC1 and TssC2 N-terminal helices (corresponding to the peptides used in this study) are highlighted by the blue frames. (**B,C**) Microscale thermophoresis interaction of the TssC1 (**B**) or TssC2 (**C**) N-terminal helical peptide with ClpV1-Nt. The MST signal change (expressed as relative fluorescence) that reflects titration of the unlabeled TssC peptide (in nM) to a constant amount of fluorescently labeled ClpV1-Nt domain was measured. Error bars represent the results from three independent experiments.

To gain further insights onto the binding and specificity of ClpV1 towards the TssC N-terminal helices, the ClpV1 N-terminal domain was purified. In addition, peptides corresponding to TssC1 (residues 23–35) and TssC2 (residues 15–29) N-terminal helices (Fig. 3A) were synthesized. Binding of the TssC peptides to ClpV1-Nt was assayed by microscale thermophoresis that allows the quantification of molecular interactions in solution. Figure 3B shows that the TssC1 peptide binds to ClpV1-Nt with a K_*D*_ of ~26 μM, a value that is comparable to the affinity measured between *V. cholerae* ClpV and the VipB (TssC) N-terminal peptide (K_*D*_ ~ 39 μM)^41^. By contrast, no binding of TssC2 peptide to ClpV1-Nt was observed (Fig. 3C).

### Structures of the ClpV1 N-terminal domain alone and in complex with the TssC1 N-terminal peptide reveal a new mode of binding

**Figure 4:**
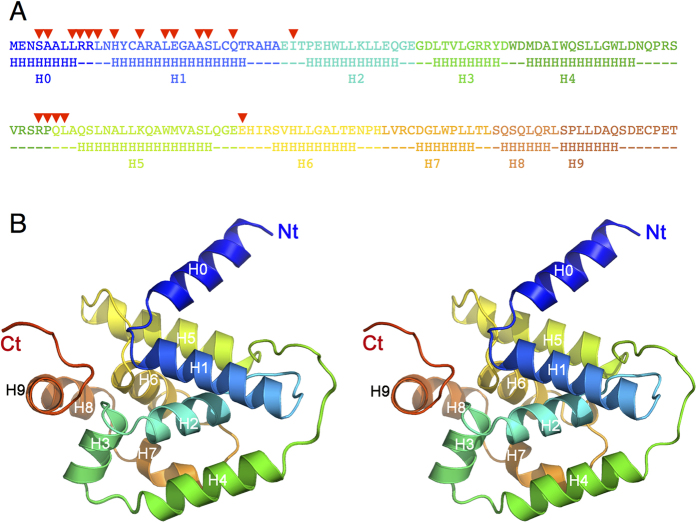
Crystal structure of the ClpV1 N-terminal domain. (**A**) Rainbow colored sequence of the ClpV1 N-terminal domain. The secondary structures are indicated below the sequence. The residues involved in the interaction with the TssC1 peptide (see Fig. 5) are indicated by the red arrowheads. (**B**) Stereoview of the ClpV1 N-terminal domain. The structure is represented as a rainbow colored ribbon. Helices are numbered H0-H8.

To obtain molecular details on the ClpV1-TssC1 interaction, we crystallized the ClpV1-Nt domain alone and in complex with the TssC1 N-terminal helix peptide. ClpV1-Nt crystallized in the P2_1_2_1_2_1_ space group and diffracted to 2.0-Å resolution (Supplemental Table S2). ClpV1-Nt structure was solved using a CsI derivative and phasing was achieved with remote SAD methods, using a 1.77-Å wavelength for data collection. The amino-acid chain could be traced between residues 1 and 163. The structure comprises 10 α-helices (H0 to H9) (Fig. 4), with overall dimensions of a flat disc of 50 × 46 × 28 Å. Interestingly, helix H0 is oriented perpendicular to helix H1, delimiting a groove.

**Figure 5:**
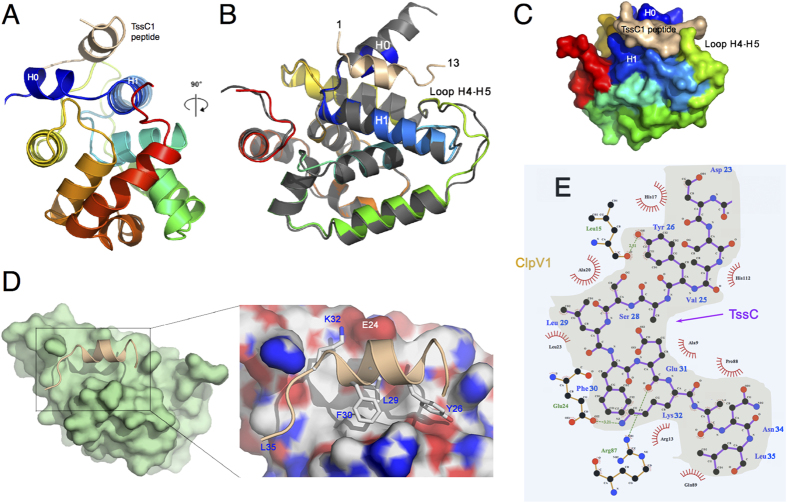
Crystal structure of the ClpV1 N-terminal domain complexed to the TssC1 N-terminal helix. (**A,B**) Ribbon view of the ClpV1-Nt/TssC1 peptide complex. The ClpV1 N-terminal domain is rainbow colored and the TssC1 peptide is shown in beige. The view in (**B**) is rotated by 90° compared to (**A**) and the uncomplexed ClpV1 N-terminal domain is superimposed and colored grey. (**C**) Surface representation of the complex. The ClpV1 N-terminal domain is rainbow colored and the TssC1 peptide is colored beige. (**D**) Surface view of the ClpV1 N-terminal domain (green) in complex with the TssC1 peptide represented as a ribbon (beige). The inset shows a close-up of the binding crevice, with the surface colored according to electrostatics (blue/red, positively/negatively charged residues; white, hydrophobic residues). (**E**) Detailed ball-and-stick scheme of the interaction. The side chains of the residues at the ClpV1-TssC1 interface of the ClpV1 N-terminal domain (blue area) are represented with brown bonds whereas that of the TssC1 peptide (grey area) are represented with violet bonds. TssC1 residues are indicated in blue whereas the side chains of ClpV1 residues involved in the interaction are indicated in green (scheme made with Ligplot+^65^).

Crystals of ClpV1-Nt in complex with the TssC1 peptide were obtained. The crystals belong to space group P2_1_2_1_2_1_ and diffracted to 2.5-Å resolution (Supplemental Table S2). The structure of the complex was determined by molecular replacement using the uncomplexed structure (Fig. 5A,B). ClpV1-Nt is defined between residues 6 and 162 in the electron density map and its structure shows that it is not subjected to significant variation compared to the unbound ClpV1-Nt domain (root mean square deviation of 0.8 Å). The TssC1 N-terminal peptide is well defined in the electron density map between the residue Asp23 and Leu35. The peptide forms a ~2-turn α-helix between Ser24 and Phe30 (SVYASLF), while the rest of the peptide (EKINL) is elongated (Fig. 5A,B). PISA analysis shows that the interaction between ClpV1-Nt and the TssC1 peptide covers 622 Å^2^ on the peptide (38% of the total surface) and 520 Å^2^ on the ClpV1 N-terminal domain (6.6% of the total surface) (Fig. 5C, Supplemental Table S3). The TssC1 peptide accommodates in the ClpV1-Nt groove delimited by helices H0 and H1, and the H4-H5 loop (Figs 4A and 5A–E, Supplemental Table S3). Most residues of the peptide are involved in the interaction, with the exception of Ser24 and Ala27. The most significant interactions involve Tyr26, Leu29, Phe30, Lys32, Ile33 and Asn34, the three last residues belonging to the elongated stretch (Fig. 5D,E, Supplemental Table S3). The co-structure of ClpV1-Nt with the N-terminal peptide of TssC1 therefore reveals a mode of binding distinct from previous co-structures^43^ (see Discussion).

### Mutagenesis studies confirm the ClpV1-TssC1 interface shown in the co-crystal

**Figure 6:**
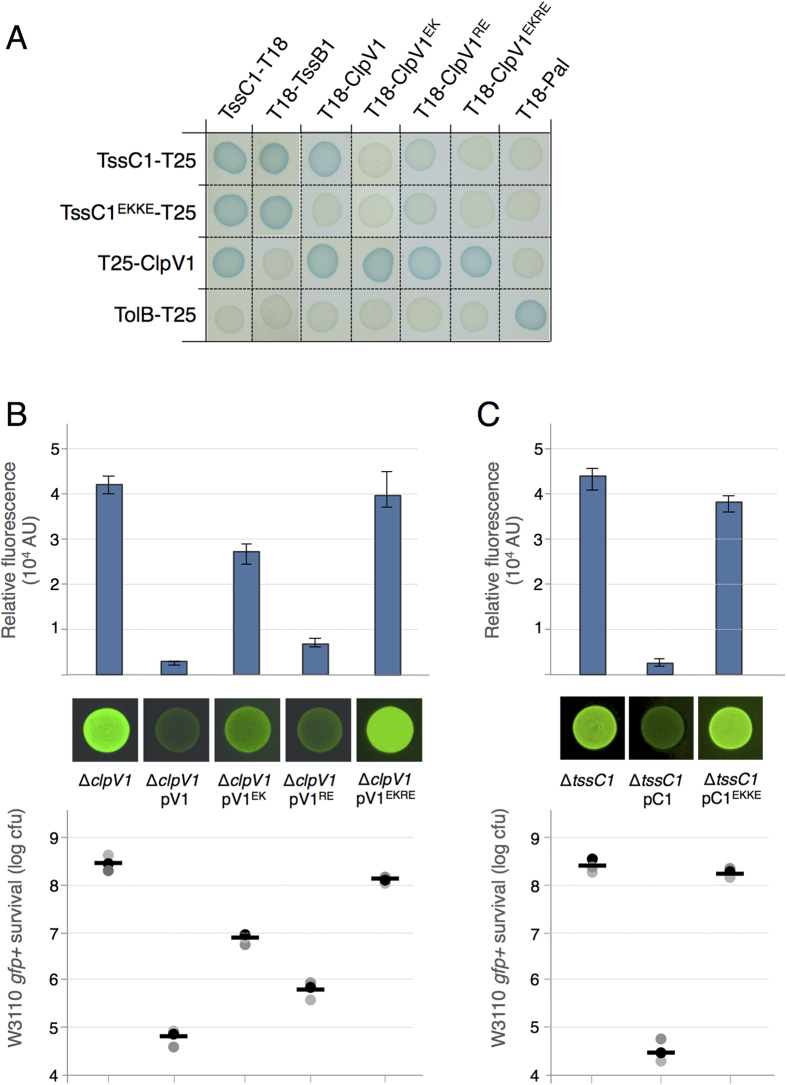
Charged residues within the ClpV1 groove and the TssC1 peptide mediate the ClpV1-TssC1 interaction. (**A**) Bacterial two-hybrid assay. BTH101 reporter cells producing the indicated wild-type or mutated proteins fused to the T18 or T25 domains of the *Bordetella* adenylate cyclase were spotted on X-Gal indicator plates. The blue color of the colony reflects the interaction between the two proteins. (**B**,**C**) Sci-1-dependent antibacterial growth inhibition. Prey cells (W3110 *gfp*^+^, kan^R^) were mixed with the indicated attacker cells, spotted onto *sci-1*-inducing medium (SIM) agar plates and incubated for 4 hours at 37 °C. The image of a representative bacterial spot is shown and the relative fluorescent levels (in arbitrary units, AU) are indicated in the upper graph. The number of recovered *E. coli* prey cells is indicated in the lower graph (in log_10_ of colony-forming units (cfu)). The circles indicate values from three independent assays, and the average is indicated by the bar.

To test the physiological relevance of the ClpV1-Nt/TssC1 peptide co-structure, we engineered point mutations within the ClpV1 groove and the TssC1 N-terminal region, and tested their effects on ClpV1-TssC1 interaction and T6SS function. The Glu24-to-Lys (ClpV1^EK^ mutant) and Arg87-to-Glu (ClpV1^RE^ mutant) substitutions (as well as the double mutant variant, ClpV1^EKRE^) were introduced in ClpV1. These mutations did not interfere with ClpV1 oligomerization (Fig. 6A). The Glu31-to-Lys and Lys32-to-Glu substitutions were introduced in TssC1 (TssC1^EKKE^ mutant). Here again, these mutations did not prevent the ability of TssC1 to oligomerize or to interact with TssB1 (Fig. 6A), two complexes previously described in EAEC^60^. However, all these substitutions affected partly or totally ClpV1-TssC1 complex formation (Fig. 6A). While the ClpV1 RE mutation had only a mild effect on the interaction, the ClpV1 EK and EKRE and the TssC1 EKKE mutations prevented formation of the ClpV1-TssC1 complex. The ClpV1 and TssC1 variants were then tested for their ability to support T6SS function. The two double mutants, ClpV1^EKRE^ and TssC1^EKKE^, did not restore the anti-bacterial activity of ∆*clpV1* (Fig. 6B) and ∆*tssC1* (Fig. 6C) EAEC cells, respectively. By contrast the ClpV1 RE and EK single substitutions caused a 9- and 310-fold decrease of the function of the EAEC Sci-1 T6SS, respectively (Fig. 6B).

## Discussion

Bacterial genomes may encode several copies of Type VI secretion gene clusters. Although these gene clusters are usually subjected to different regulatory controls and expressed in different conditions, distinct Type VI secretion machineries may co-exist in the same bacterial cell^1^,^2^. There is therefore a need to control proper assembly of these machines and to allow recruitment of cognate subunits. Indeed, previous studies have demonstrated that specificity operates during machine assembly, more specifically for the interaction between the inner tube Hcp subunit and its cognate TssB sheath protein in enteroaggregative *E. coli*^21^. Here, we show that a second level of specificity exists, at the stage of sheath disassembly/recycling. The EAEC 17-2 strain genome encodes two T6SS gene clusters^44^,^58^, each containing its own ClpV ATPase. These two proteins share 38/65% sequence identity/similarity (Supplemental Figure S1). Here, we show that these two proteins are not interchangeable and that ClpV2 cannot rescue the anti-bacterial defects of a ∆*clpV1* strain, and vice versa. Using a combination of bacterial two-hybrid, co-immunoprecipitation and *in vitro* microscale thermophoresis assays, we provide evidence that specificity is dictated by binding of the TssC N-terminal helix to the ClpV N-terminal domain. The N-terminal regions of TssC1 and TssC2 (residues 1–163) are much more divergent (20/53% identity/similarity) than full-length proteins (38/65%) (Supplemental Figure S1). The crystal structure of the ClpV1 N-terminal domain in complex with the TssC1 N-terminal helix further showed that the TssC1 helix inserts into a cleft delimitated by the H0 and H1 helices and the H4-H5 loop (Figs 4A, 5 and 6, Supplemental Table S3). Interestingly, binding of the TssC1 N-terminal helix to ClpV1-Nt does not cause a significant conformational change within ClpV. This situation is similar to that reported for the *V. cholerae* ClpV_Vc_-VipB complex (see below)^41^. The weak K_*D*_ value (26 μM) is also very similar to that measured in *V. cholerae* (39 μM)^41^. However, the K_*D*_ values obtained *in vitro* might not represent the *in vivo* situation. *In vivo*, it is assumed that the ClpV ATPase is recruited to the contracted sheath^15^,^18^,^22^,^61^, and therefore hundreds of TssC N-terminal helices are available, probably resulting in avidity higher by several orders of magnitudes as compared to the affinity of isolated fragments.

**Figure 7:**
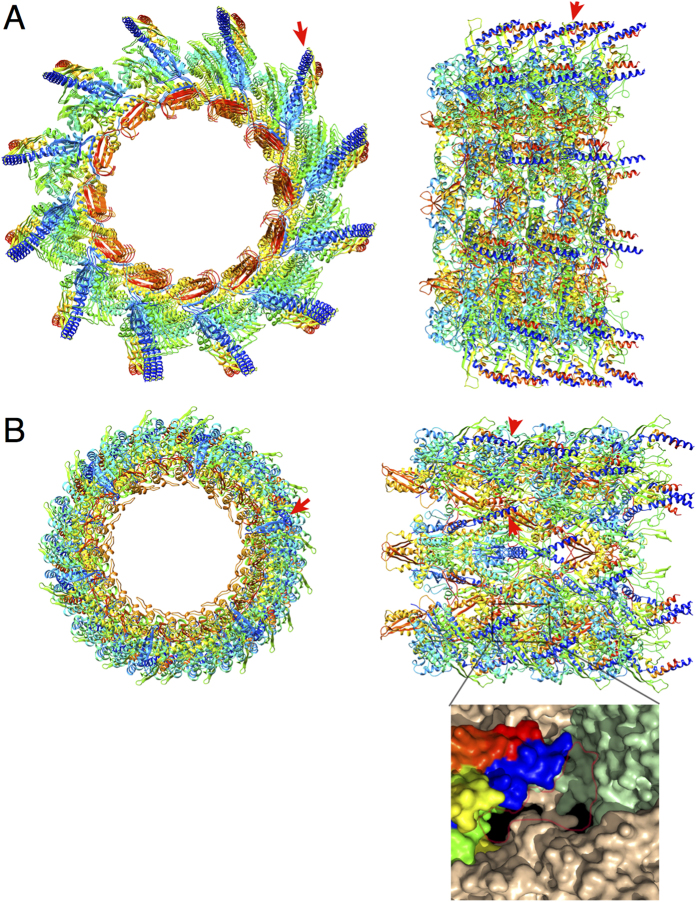
Position of TssC N-terminal helix in the contracted and extended T6SS sheath models. (**A**) Contracted *V. cholerae* VipAB sheath atomic structure^24^ (right, top view; left, side view). Four sheath rows are shown (right, top view; left, side view). The red arrows indicate the VipB (TssC) first visible residue. (**B**) Extended *V. cholerae* VipAB sheath molecular model. The inset shows a close-up of the VipB N-terminus and the flanking cavity (surligned in red) that could accommodate the VipB 1-60 fragment.

The structure of the T6SS contracted sheath^23^,^24^,^62^ revealed that the TssC N-terminal helices are not visible in the electron density map, probably due to their high flexibility. However, the position of the first visible TssC residue (residue 61, pointed by the red arrow in Fig. 7A) strongly suggests that the N-terminal VipB/TssC helix protrudes far out of the sheath cylinder and hence might be accessible to ClpV. This observation supports the current model in which sheath contraction extricates the TssC N-terminal helix, leading to ClpV recruitment and sheath subunits recycling^18^,^22^. However, this model assumes that the TssC N-terminal helix is buried within the extended sheath. To date, the molecular structure of the T6SS sheath under the extended conformation is not available. In a recent study, a low resolution model of the extended *V. cholerae* VipAB sheath was modeled using the low resolution EM map of the extended T4 phage tail sheath^23^. By superimposing the VipAB EM map to the gp18 T4 phage sheath protein, gross features of the sheath structure were obtained^23^. To obtain high-resolution details, we applied this approach by fitting the recently released VipAB molecular model into the extended T4 phage tail sheath. In the extended model, VipB residue 61 is also located near the sheath surface, but packed against the cylinder surface (red arrows in Fig. 7B). We noticed an empty cavity nearby residue 61 that is large enough to accommodate the VipB N-terminal segment 1–60 (Fig. 7B). In this configuration, the TssC N-terminal helix will be buried and therefore not accessible to ClpV.

**Figure 8:**
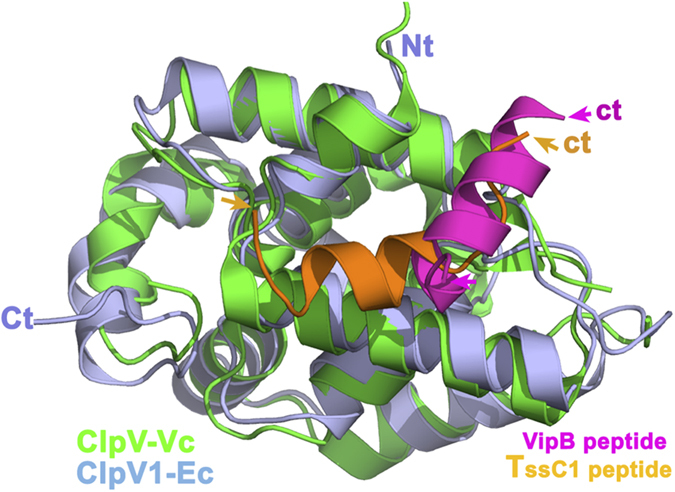
Structural comparison between the EAEC and *Vibrio cholerae* ClpV N-terminal domains in complex with TssC/VipB peptides. Ribbon view of the enteroaggregative *E. coli* ClpV1-Nt/TssC1 peptide (ClpV in blue, peptide in orange; PDB 4HH6) superimposed to the *V. cholerae* ClpV-Nt/VipB peptide (ClpV in green, peptide in pink; PDB 3ZRJ^41^).

The structures of the *V. cholerae* (PDB 3ZRI)^41^ and EAEC (this study) ClpV N-terminal domains share very close folds (Fig. 8). They display an elongated crevice at the same location, in which the TssC (VipB) helix inserts. While the overall scheme is the same, one can notice several differences. First, the position and orientation of the *V. cholerae* VipB and EAEC TssC1 N-terminal α-helices within the groove are slightly different. The TssC1 peptide starts with an alpha helical structure followed by an extended segment (Fig. 8). By contrast, the VipB peptide is entirely helical and locates within ClpV_Vc_ at a position that is occupied by the TssC1 extended segment in ClpV1 (Fig. 8). This indicates, as already suggested^43^, that although ClpV exhibits a common fold in different T6SS, the nature and position of residues involved in TssC binding vary significantly enough to elicit variations in the mode of interaction. In addition, the ClpV1 groove that accommodates the TssC1 helix is constituted of a hydrophobic central area borded by charged residues, Glu24 of H1 in contact with the Lys29 residue of TssC1, and Arg87 of the H4-H5 loop in contact with a main-chain carboxylic group of TssC1 (Fig. 5D,E). It has been proposed that ClpV proteins could be categorized in two phylogenetic groups differing in the nature of residues within the groove (charged or uncharged residues)^43^. It has also been suggested that ClpV proteins with charged grooves cannot bind TssC N-terminal helices and require the assistance of an accessory protein, TagJ^43^,^63^. However, although the EAEC ClpV1 protein presents a central hydrophobic groove borded by charged residues, (i) it is able to bind directly to the TssC N-terminal helix, (ii) the ClpV1 charged residues are critical for the ClpV1-TssC1 interaction, and (ii) no TagJ homologue is encoded within the EAEC genome. These observations suggest that interaction between ClpV1 and TssC1 in EAEC represents a third mode of binding dependent on a partially charged interface but independent on TagJ. This variability in the mechanism of ClpV binding to TssC supports the notion that specific determinants regulate recruitment of ClpV to contracted sheaths in cells with distinct T6SS machines.

## Additional Information

**How to cite this article**: Douzi, B. *et al*. Structure and specificity of the Type VI secretion system ClpV-TssC interaction in enteroaggregative *Escherichia coli. Sci. Rep.* **6**, 34405; doi: 10.1038/srep34405 (2016).

## Supplementary Material

Supplementary Information
