# Supplementary material for: Structure and specificity of the Type VI secretion system ClpV-TssC interaction in enteroaggregative Escherichia coli
Source: Sci Rep. 2016 Oct 4;6:34405. doi: 10.1038/srep34405 (PMC5048182; doi:10.1038/srep34405)
Supplement: Supplementary Information [file srep34405-s1.pdf]

SUPPLEMENTAL DATA

Structure and specificity of the Type VI secretion system ClpV-TssC interaction in enteroaggregative *Escherichia coli*.

B. Douzi, Y.R. Brunet, S. Spinelli, V. Lensi, P. Legrand, S. Blangy,
A. Kumar, L. Journet, E. Cascales & C. Cambillau

Supplemental Figure S1. Clustal W alignment of the EAEC ClpV1 and ClpV2 amino-acid sequences.

The two full-length protein sequences were aligned using ClustalW on the Pôle Rhone-Alpes de BioInformatique (PRABI) server (https://npsa-prabi.ibcp.fr/cgi-bin/npsa_automat.pl?page=/NPSA/npsa_clustalw.html). Identical residues are shown in red and indicated by a star. Similar residues are shown in blue or green. The red arrow indicates the position of the ClpV N-terminal domain C-terminus used in all the constructs.

```
                   10        20        30        40        50        60        70        80        90       100
                    |         |         |         |         |         |         |         |         |         |
ClpV1      VNNMENSAALLRRLNHYCARALEGAASLCQTRAHAEITPEHWLLKLLEQGEGDLTVLGRRYDWDMDAIWQSLLGWLDNQPRSVRSRPQLAQSLNALLKQA
ClpV2      --MSIYLKPIINKLTPESRNTLDSAINYAISRSHHEVDCLHLLWKLLQEHKYIAEVLYEQSLFNPEWVLNAIESELIRINTVPQSSPVFSESMQTLLEKT
                   .::.:*.  . .:*:.* . . :*:* *:   * * ***:: :    ** .:  :: : : ::: . * .     :* * :::*:::**:::

                  110       120       130       140       150       160       170       180       190       200
                    |         |         |         |         |         |         |         |         |         |
ClpV1      WMVASLQGE-EHIRSVHLLGALTENPHLVRCDGLWPLLTLSQSQLQRLSPLLDAQSDECPETLQDAEPVLPQGDSVTFIGRPVGADTAGIPSGDLPPVLQ
ClpV2      WIHASTKWQIDHIDIPVFLSTMIN--FR---DSIFPLN---------VSDALCCDMDVAEELLISFSDEAEHSASH----RPT-------DRSS-----H
           *: ** : : :**    :*.:: :  .    *.::**          :*  * .: * . * * . .    :. *     **.         ..     :

                  210       220       230       240       250       260       270       280       290       300
                    |         |         |         |         |         |         |         |         |         |
ClpV1      GALDKFTRDITASAREGKIDPVSGRDTEIRQMVDILSRRRKNNPILVGDPGVGKTALVEGLALRIVEGNVPESLRPVTLRTLDLGLLQAGAGVKGEFEQR
ClpV2      EYLSKYTENLSLRAETGKLDPVTGREKEVRQLIDILLRRRQNNPILTGEPGVGKSSIVEGLALQIASGRVPDVLKNVRIHALDMGALLAGASVRGEFENR
             *.*:*.:::  *. **:***:**:.*:**::*** ***:*****.*:*****:::******:*..*.**: *: * :::**:* * ***.*:****:*

                  310       320       330       340       350       360       370       380       390       400
                    |         |         |         |         |         |         |         |         |         |
ClpV1      LKNVIDAVQLSPAPVLLFIDEAHTLIGAGNQAGGADAANLLKPALARGELRTIAATTWSEYKQYLERDAALERRFQMVKVDEPDDETACLMLRSLKSRYA
ClpV2      LKSLLTELNSLDGTAILFIDEAHSLIGAGGLPGQTDAANLLKPALARGELRIIAATTWGEYKKYFEKDGALARRFQIVKVAEPNQDVTAEMLRSLLPMME
           **.::  ::   ...:*******:*****. .* :**************** ******.***:*:*:*.** ****:*** **:::.:. ***** .
```

```
                    410       420       430       440       450       460       470       480       490       500
                      |         |         |         |         |         |         |         |         |         |
ClpV1        EHHNVHITDEAVRAAVTLSRRYLTERQLPDKAVDLLDTAAARVRMSLDTVPEQLTRIRSQLASLGMEKQALLEDIAVGHQNHGERLSAIEQEENVLMTAR
ClpV2        KHHNVSIREEAITATVHLSDRYLHGRRQPDKSVSLLDTACSRVAVSQSTSPDAIQDVEASLVRYQGELALLTQ-----ERSDVLRQEMLANKIAQLEEEL
             :**** * :**: *:* ** ***  *: ***:*.*****.:** :* .* *: :  :.:.*.    *   * :     .:..  * . : ::   *

                    510       520       530       540       550       560       570       580       590       600
                      |         |         |         |         |         |         |         |         |         |
ClpV1        DDLEQQYARECELTGELLESRSDISRQSETHHLQQALHDIQQNQPLLSVDVDVRTVAGVVADWTGVPLSSLMKDEQTELLHLEKDIGRRVVGQDVALESI
ClpV2        EQLKSAWRHQSELVAKIQSS-DDIS--SKNMYRKELESAYKKDSPMVFECVDKNCVADVVSGWTGVPLGVCLDGEQQKASGLLRCLEQRVLGQRYAMSAI
             ::*:. : ::.**..:: .* .***  *:. : ::     :::.*::   ** . **.**:.******.  :..** :   * : : :**:**  *:.:*

                    610       620       630       640       650       660       670       680       690       700
                      |         |         |         |         |         |         |         |         |         |
ClpV1        AQRLRAAKTGLTSGNGPQEVFLLVGPSGVGKTETALALADVMYGGEKSLITINLSEYQEPHTVSQLKGSPPGYVGYGQGGILTEAVRKRPYSVVLLDEVE
ClpV2        ASQVLICRADLKDPVKPDGVFLLAGPSGTGKTETARALAEFVYGDENKLITINMTEFQEAHTVSTLKGAPPGYVGFGQGGTLTERVSHNPYSVILLDEIE
             *.::  .::.*..   *: ****.****.****** ***:.:**.*:.*****::*:**.**** ***:******:**** *** * :.****:****:*

                    710       720       730       740       750       760       770       780       790       800
                      |         |         |         |         |         |         |         |         |         |
ClpV1        KAHRDVLNLFYQVFDRGFMRDGEGREIDFRNTVILMTSNLGSDLLMQQLSEKPETTESELHELIRPLLRDHFQPALLARFQTVIYRPLTPSAMRTIVEMK
ClpV2        KAHPDVLEFFFQIFDSGIIEDAEGKMVSFRDCLIIMTSNFASENITNIWNDG-ETNRDRIKEMLLPLFNEHFGAAFMGRTNLIPFTPLHSKTLRDIVLIK
             *** ***::*:*:** *::.*.**: :.**: :*:****:.*: : :  .:  **....::*:: **:.:** .*::.* : : : ** ..::* ** :*

                    810       820       830       840       850       860       870       880       890
                      |         |         |         |         |         |         |         |         |
ClpV1        LAQVCERLHCHYG--LSTSVDERVYDALTSACLLPDTGARNVESLLNQQLLPVLSRQLLSHMAAKQKPQALALAWSDEDGMVIELRQECAL-
ClpV2        IDKICQRFEQASGQMYKIEYNDSLIDWITHHCQCDKSGARDIDSVLNSTVLPVLAR-YLTDSEDNRTPKKIRISVR-KNNITLRSSQFATRN
             : ::*:*:.   *   . . :: : * :*  *   .:***:::*:**. :****:*  *:.   ::.*: : ::   ::.:.:.  * .:
```

Supplemental Table S1. Strains, plasmids and oligonucleotides used in this study.

Strains

Strains	Description and genotype	Source
E. coli K-12		
DH5α	F-, Δ(*argF-lac*)U169, *phoA*, *supE44*, Δ(lacZ)M15, *relA*, *endA*, *thi*, *hsdR*	New England Biolabs
W3110	F-, lambda- IN(*rrnD-rrnE*)1 *rph*-1	Laboratory collection
BTH101	F-, *cya-99, araD139, galE15, galK16, rpsL1 (Str^R), hsdR2, mcrA1, mcrB1.*	Karimova et al., 2005
BL21(DE3) pLys	F-, miniF *lysY lacI^q* (Cm^R) / *fhuA2 lacZ::T7 gene1 [lon] ompT gal sulA11 R(mcr-73::miniTn10*--Tet^S*)2 [dcm] R(zgb-210::Tn10*--Tet^S*) endA1 Δ(mcrC-mrr) 114::IS10*	New England Biolabs
Enteroaggregative *E. coli*		
17-2	WT enteroaggregative *Escherichia coli*	Arlette Darfeuille-Michaud
17-2Δ*clpV1*	17-2 deleted of the *clpV* gene of the *sci1* T6SS gene cluster (EC042_4530)	Brunet et al., 2015
17-2Δ*clpV2*	17-2 deleted of the *clpV* gene of the *sci2* T6SS gene cluster (EC042_4577)	M.S. Aschtgen
17-2Δ*tssC1*	17-2 deleted of the *tssC* gene of the *sci1* T6SS gene cluster (EC042_4525)	Brunet et al., 2015

Plasmids

Vectors	Description	Source
Expression vectors		
pUA66-rrnb	P_{rrnB} *::gfpmut2* transcriptional fusion in pUA66	Zaslaver et al., 2006
pASK-IBA37	cloning vector, P*tet*, Amp^R	IBA Technology
pIBA37-ClpV1$_{FLAG}$	*sci1 clpV*, C-terminal FLAG tag cloned into pASK-IBA37	This study

pIBA37-ClpV1-Nt_{FLAG}	*sci1 clpV* N-terminal fragment (residues 1-162), C-terminal FLAG tag cloned into pASK-IBA37(+)	This study
pIBA37-ClpV1-$E24K_{FLAG}$	*clpV1* Glu24-to-Lys substitution into pIBA37-$ClpV1_{FLAG}$	This study
pIBA37-ClpV1-$R87E_{FLAG}$	*clpV1* Arg87-to-Glu substitution into pIBA37-$ClpV1_{FLAG}$	This study
pIBA37-ClpV1-E24K-$R87E_{FLAG}$	*clpV1* Glu24-to-Lys and Arg87-to-Glu substitutions into pIBA37-$ClpV1_{FLAG}$	This study
pIBA37-$ClpV2_{FLAG}$	*sci2 clpV*, C-terminal FLAG tag cloned into pASK-IBA37	This study
pIBA37-ClpV2-Nt_{FLAG}	*sci2 clpV* N-terminal fragment (residues 1-147), C-terminal FLAG tag cloned into pASK-IBA37(+)	This study
pBAD33	cloning vector, pACYC184 origin, *Para*, *araC* Cm^R	Guzman et al., 1995
pBAD33-$TssC1_{VSV-G}$	*sci1 tssC*, C-terminal VSV-G tag cloned into pBAD33	This study
pBAD33-TssC1-E31K-$K32E_{VSV-G}$	*tssC1* Glu31-to-Lys and Lys32-to-Glu substitutions into pBAD33-$TssC1_{VSV-G}$	This study
pBAD33-$TssC1\Delta h_{VSV-G}$	Deletion of amino-acids 23-35 of *sci1 tssC* into pBAD33-$TssC1_{VSV-G}$	This study
pBAD33-$TssC2_{VSV-G}$	*sci2 tssC*, C-terminal VSV-G tag cloned into pBAD33	This study
pBAD33-$TssC2\Delta h_{VSV-G}$	Deletion of amino-acids 2-15 of *sci2 tssC* into pBAD33-$TssC2_{VSV-G}$	This study

Bacterial Two-Hybrid vectors

pT18-FLAG	Bacterial Two Hybrid vector, ColE1 origin, P*lac*, T18 fragment of *Bordetella pertussis* CyaA, Amp^R	Battesti & Bouveret, 2008
pT18-Pal	Soluble region of *E. coli* K-12 Pal cloned downstream T18 in pT18-FLAG	Battesti & Bouveret, 2008
pClpV1-T18	*sci1 clpV* cloned upstream T18 into pT18-FLAG	This study
pT18-ClpV1	*sci1 clpV* cloned downstream T18 into pT18-FLAG	This study
pT18-ClpV1-E24K	*clpV1* Glu24-to-Lys substitution into pT18-ClpV1	This study
pT18-ClpV1-R87E	*clpV1* Arg87-to-Glu substitution into pT18-ClpV1	This study
pT18-ClpV1-E24K-R87E	*clpV1* Glu24-to-Lys and Arg87-to-Glu substitutions into pT18-ClpV1	This study
pT18-ClpV2	*sci2 clpV* cloned downstream T18 into pT18-FLAG	This study
pT25-FLAG	Bacterial Two Hybrid vector, p15A origin, P*lac*, T25 fragment of *Bordetella pertussis* CyaA, Kan^R	Battesti & Bouveret, 2008
pTolB-T25	*E. coli* K-12 *tolB* cloned upstream T25 in pT25-FLAG	Battesti & Bouveret, 2008
pClpV1-T25	*sci1 clpV* cloned upstream T25 into pT25-FLAG	This study
pT25-ClpV1	*sci1 clpV* cloned downstream T25 into pT25-FLAG	This study
pTssC1-T25	*sci1 tssC* cloned upstream T25 into pT25-FLAG	Zoued et al., 2013
pTssC1-E31K-K32E-T25	*tssC1* Glu31-to-Lys and Lys32-to-Glu substitutions into pTssC1-T25	This study
pTssC2-T25	*sci2 tssC* (EC042_4564) cloned upstream T25 into pT25-FLAG	This study

All others BACTH constructs have been described in Zoued et al., 2013.

Oligonucleotides

Name	Destination	Sequence (5' to 3’)
For plasmid construction [a,b,c]		
5-pIBA37-ClpV1$_{FLAG}$	pIBA-ClpV1$_{FLAG}$ / pIBA-ClpV1-Nt	GACAAAAATCTAGAAATAATTTTGTTTAACTTTAAGAAGGAGATATACAAATGAATAACATGGAAAATTCGGCAGCCCTGTTACG
3-pIBA37-ClpV1$_{FLAG}$	pIBA-ClpV1$_{FLAG}$	GATGGTGATGGTGATGCGATCCTCTGCTAGCTTA*TTTATCATCGTCGTCTTTATAATC*TAACGCGCATTCCTGCCGCAGCTC
3- pIBA37-ClpV1-Nter	pIBA-ClpV1-Nt	GATGGTGATGGTGATGCGATCCTCTGCTAGCTTA*TTTATCATCGTCGTCTTTATAATC*TAACGTCTCCGGACACTCATCAGACTG
5-pIBA37-ClpV2$_{FLAG}$	pIBA-ClpV2$_{FLAG}$ / pIBA-ClpV2-Nt	GACAAAAATCTAGAAATAATTTTGTTTAACTTTAAGAAGGAGATATACAAATGGTGAGTATCTATCTGAAACCAATTATTAATAAATTAACTCCAGAAAG
3- pIBA37-ClpV2$_{FLAG}$	pIBA-ClpV2	GATGGTGATGGTGATGCGATCCTCTGCTAGCTTA*TTTATCATCGTCGTCTTTATAATC*ATTACGAGTTGCAAATTGCGAACTACGTAATGTAATG
3-pIBA37-ClpV2-Nter	pIBA-ClpV2-Nt	GATGGTGATGGTGATGCGATCCTCTGCTAGCTTA*TTTATCATCGTCGTCTTTATAATC*AAGTAACTCCTCAGCTACATCCATATCACAACAT
5-pBAD33-TssC1$_{VSV-G}$	pBAD33-TssC1$_{VSV-G}$	CTCTCTACTGTTTCTCCATACCCGTTTTTTTGGGCTAGC*AGGAGGTATTACACC*ATGCTGATGTCTGTACAGAAAGAAAAGAACGTTG
3-pBAD33-TssC1$_{VSV-G}$	pBAD33-TssC1$_{VSV-G}$	GGTCGACTCTAGAGGATCCCCGGGTACCTTA*TTTTCCTAATCTATTCATTTCAATATCTGTATA*CGCTTTTGCCTTCGGCATCTGCG
5-pBAD33-TssC2$_{VSV-G}$	pBAD33-TssC2$_{VSV-G}$	CTCTCTACTGTTTCTCCATACCCGTTTTTTTGGGCTAGC*AGGAGGTATTACACC*ATGACAGTTGCATCAACATTAGG
3-pBAD33-TssC2$_{VSV-G}$	pBAD33-TssC2$_{VSV-G}$	GGTCGACTCTAGAGGATCCCCGGGTACCTTA*TTTTCCTAATCTATTCATTTCAATATCTGTATA*ACTTACACTTGATGGTAAATCAGC
T25T18C-5-4530	pClpV1-T18 / pClpV1-T25	CGGATAACAATTTCACACAGGAAACAGCTATGACCATGAATAACATGGAAAATTCGGCAGCCCT
T18C-3-4530	pClpV1-T18	CCTCGCTGGCGGCTAAGCTTGGCGTAATTAACGCGCATTCCTGCCGCAG
T25C-3-4530	pClpV1-T25	GTTTGCGTAACCAGCCTGATGCGATTGCTGTAACGCGCATTCCTGCCGCAG
T25N-5-4530	pT25-ClpV1	GGCGGGCTGCAGATTATAAAGATGACGATGACAAGAATAACATGGAAAATTCGGCAGCCC
T18N-5-4530	pT18-ClpV1	CGCCACTGCAGGGATTATAAAGATGACGATGACAAGAATAACATGGAAAATTCGGCAGCCCT
T25T18N-3-4530	pT18-ClpV1 / pT25-ClpV1	CGAGGTCGACGGTATCGATAAGCTTGATATCGAATTCTAGTTATAACGCGCATTCCTGCCGCAG
T25T18C-5-TssC2	pTssC2-T25	CGGATAACAATTTCACACAGGAAACAGCTATGACCATGACAGTTGCATCAACATTAGG

T25C-3-TssC2	pT25-TssC2	GTTTGCGTAACCAGCCTGATGCGATTGCTGACTTACACTTGATGGTAAATCAGC
5- pETG-ClpV1-Nter	pETG20A-ClpV1-Nt	GGGGACAAGTTTGTACAAAAAAGCAGGCTTAGAAAACCTGTACTTCCAGGGTGTGAATAA CATGGAAAATTC
3- pETG-ClpV1-Nter	pETG20A-ClpV1-Nt	GGACCACTTTGTACAAGAAAGCTGGGTCTTATTATAACGTCTCCGGACACTCA
For site-directed mutagenesis [d,e,f]		
A-C1-Δh	pBAD33-TssC1$_{VSV-G}$	GTGGTATCTGAAGCGCATGCC*GGCAGT*CCGGTATCTGCCCTGAGTGCAC
A-C1-Δh	pBAD33-TssC1$_{VSV-G}$	GTGCACTCAGGGCAGATACCGG*ACTGCC*GGCATGCGCTTCAGATACCAC
A-C2-Δh	pBAD33-TssC2$_{VSV-G}$	AGCAGGAGGTATTACACC*ATGACA*GATGATTGTCTTGAAGAG
A-C2-Δh	pBAD33-TssC2$_{VSV-G}$	CTCTTCAAGACAATCATC*TGTCAT*GGTGTAATACCTCCTGCT
A-ClpV1-E24K	pIBA37-ClpV1$_{FLAG}$ and pT18-ClpV1	CTGTGCCCGTGCACTG**A**AAGGCGCAGCCTCCCTTTG
B-ClpV1-E24K	pIBA37-ClpV1$_{FLAG}$ and pT18-ClpV1	CAAAGGGAGGCTGCGCCTT**T**CAGTGCACGGGCACAG
A-ClpV1-R87E	pIBA37-ClpV1$_{FLAG}$ and pT18-ClpV1	CCGTAGCGTACGCAGT**GAA**CCGCAGCTTGCGCAGTC
B-ClpV1-R87E	pIBA37-ClpV1$_{FLAG}$ and pT18-ClpV1	GACTGCGCAAGCTGCGG**TTC**ACTGCGTACGCTACGG
A-TssC1-E31K-K32E	pBAD33-TssC1$_{VSV-G}$ and pTssC1-T25	GTATATGCTTCCCTGTTT**A**AA**G**AAATTAACCTGAGTCCGG
B-TssC1-E31K-K32E	pBAD33-TssC1$_{VSV-G}$ and pTssC1-T25	CCGGACTCAGGTTAATTT**C**TT**T**AAACAGGGAAGCATATAC

[a] Sequence annealing on the target plasmid underlined.
[b] FLAG or VSV-G epitope coding sequence *italicized*.
[c] Additional Shine Dalgarno sequence *italicized underlined*.
[d] Codons upstream and downstream the deletion *italicized*.
[e] Nucleotide substitutions in **bold.**
[d] Mutagenized codons underlined**.**

Supplemental Table S2. Data collection and refinement statistics

**DATA COLLECTION**	Soleil Proxima 1	Soleil Proxima 1
PDB	4HH5	4HH6
Proteins	ClpV1-Nt	ClpV1-Nt/TssC1-pept
Space group, cell (Å,°)	$P2_12_12_1$, 40.9, 58.7, 65.6	$P2_12_12_1$, 40.9, 46.85, 75.5
Resolution limits[a] (Å)	50.0-**2.0** (2.05-2.0)	50-**2.5** (2.56-2.5)
Rmeas[a] (%)	5.2 (38)	8.6 (72.3)
Nr. of observations[a,b]	146934 (9916)	29258 (1739)
Nr. unique reflections[a,b]	19805 (1405)	5360 (388)
Mean((I)/sd(I)) [a]	27.1 (5.5)	13.1 (2.2)
Completeness[a] (%)	96.1 (92.1)	99.6 (98.5)
Multiplicity[a]		
**REFINEMENT**		
Resolution[a] (Å)	43.8-2.0 (2.24-2.0)	36.0-**2.5** (2.79-2.5)
Nr of reflections[a]	10703(2770)	5359 (1358)
Atoms : protein, ions, water	1275 / 1 /107	1297 / 27
Nr test set reflections	512	516
R_{work}/R_{free}[a] (%)	0.186/0.205 (0.181/0.234)	0.244/0.263 (28.1/32.1)
r.m.s.d.bonds (Å) / angles (°)	0.008 / 1.04	0.008 / 1.36
B-wilson / B-average (Å^2)	28.35 / 29.6	57.6 / 55
Ramachandran: preferred/allowed (%)	97.5 / 2.5	91.6 / 6.0 / 2.4

[a] numbers into parenthesis refer to the highest resolution bin.

[b] Friedel pairs not merged

Supplemental Table S3. Interaction between the ClpV1-N-terminal domain and the TssC1 peptide.
(**A**) Analysis of the interaction surface. (**B**) Residues of TssC1 peptide interacting with the ClpV1-N-terminal domain. (**C**) Residues of the ClpV1-N-terminal domain interacting with the TssC1 peptide. ASA : accessible surface area. BSA : buried surface area. Analysis performed with PISA.

A

	Peptide 1		ClpV-Nt	
**Interface Å²**	622.5	38.4%	520.8	6.6%
**Total surface Å²**	1620.9		7912.5	

B

Peptide 1	ASA	BSA
ASP 23	70.21	24.4
VAL 25	72.85	20.3
TYR 26	133.51	113.0
SER 28	59.69	19.7
LEU 29	118.81	113.0
PHE 30	130.83	62.7
GLU 31	131.80	22.1
LYS 32	142.15	101.2
ILE 33	97.17	75.5
ASN 34	114.88	57.5
LEU 35	230.48	12.6

C

ClpV1-Nt	ASA	BSA
SER 8	31.16	10.5
ALA 9	67.64	25.9
LEU 12	57.58	52.7
ARG 13	182.26	46.3
LEU 15	10.34	10.3
HIS 17	169.33	52.7
ALA 20	41.75	38.7
LEU 23	13.70	13.5
GLU 24	109.71	68.4
ALA 27	26.96	26.8
SER 28	55.93	7.2
GLN 31	32.77	2.5
ILE 38	5.36	4.2
ARG 87	215.54	77.3
PRO 88	31.75	28.6
GLN 89	64.68	27.6
LEU 90	41.49	18.1
